# *Acinetobacter baumannii* during COVID-19: What Is the Real Pandemic?

**DOI:** 10.3390/pathogens12010041

**Published:** 2022-12-27

**Authors:** Karyne Rangel, Salvatore Giovanni De-Simone

**Affiliations:** 1 Center for Technological Development in Health (CDTS), National Institute of Science and Technology for Innovation in Neglected Population Diseases (INCT-IDPN), Oswaldo Cruz Foundation (FIOCRUZ), Rio de Janeiro 21040-900, Brazil; 2 Laboratory of Epidemiology and Molecular Systematics (LESM), Oswaldo Cruz Institute, Oswaldo Cruz Foundation (FIOCRUZ), Rio de Janeiro 21040-900, Brazil; 3 Post-Graduation Program in Science and Biotechnology, Department of Molecular and Cellular Biology, Biology Institute, Federal Fluminense University (UFF), Niterói 22040-036, Brazil

The novel Coronavirus Disease 2019 (COVID-19), caused by the Severe Acute Respiratory Syndrome Coronavirus 2 (SARS-CoV-2) pandemic, has had a monumental impact on public health globally. Adding to this impact are SARS-CoV-2 bacterial coinfections and/or secondary infections that have become the hidden threats behind COVID-19, such as those caused by *Acinetobacter baumannii* (all infections). These other types of infection have increased the severity and risks associated with SARS-CoV-2 infection, playing a major role in SARS-CoV-2 morbidity and mortality of patients [1]. The prevalence of coinfection and secondary infections, in patients with SARS-CoV-2 infection, has been reported to range globally from 0.6 to 45% [2]. According to recent studies, bacterial coinfection upon admission has been reported in 3.1–3.5% of COVID-19 patients, while secondary bacterial infections, following hospitalization, occurred in up to 15% of patients [2,3,4]. During the SARS-CoV-2 pandemic, a study from Wuhan, China, demonstrated that *A. baumannii* was the most common bacterial secondary infection, with 91.2% of isolates resistant to carbapenem [5]. Additionally, multidrug-resistant (MDR) *A. baumannii* infections were reported as one of the most prevalent bacterial coinfections in patients with COVID-19 [6,7].

Throughout the COVID-19 pandemic, the challenges faced by healthcare professionals were further exacerbated by multiple outbreaks of MDR and extensively drug-resistant (XDR) *A. baumannii* [8,9]. Compared to the pre-pandemic era, MDR *A. baumannii* infections occured more frequently during the COVID-19 pandemic [10]. Among patients with COVID-19, 5–15% have moderate or severe symptoms and require hospitalization, and some require intensive care unit (ICU) follow-up [11]. Certain risk factors increased the incidence of *A. baumannii* infection during the COVID-19 pandemic, and were linked to invasive and non-invasive mechanical ventilation, industrial oxygen administration, invasive procedures, and prolonged hospital stay [12]. Recent reports suggest that up to 80% of ICU-admitted COVID-19 patients require invasive mechanical ventilation [13]. Among these patients, MDR *A. baumannii* has been identified as a frequent cause of secondary bacterial infection, associated with a two-fold increase in COVID-19-related mortality [1]. Another study identified *A. baumannii* as the second most prevalent secondary disease causative agent, displaying XDR [14]. Previous studies in Brazil showed a high incidence of secondary infections, mainly caused by MDR *A. baumanni* (96%). These bacteria were also associated with longer ICU stay, mechanical ventilation use, and higher mortality [15]. *A. baumannii* was present in blood cultures in 27.5% of cases, and in respiratory cultures of patients diagnosed with SARS-CoV-2, in 33.3% of cases [16,17]. Due to the high prevalence of antibiotic resistance, genomic plasticity, and successful virulence mechanisms of *A. baumannii*, we must study the co-occurrence of COVID-19 and *A. baumannii*; in particular, we must look at their association and clinical outcome, as the negative prognosis of this coinfection may be rapid in its onset [18].

As we join efforts to prevent and control the COVID-19 pandemic, we must be aware of the impacts of coinfections and secondary infections with antibiotic-resistant *A. baumannii* on worsening health outcomes. We should be more diligent and ensure we are administering antibiotics effectively, in order to hopefully reduce antibiotic resistance, with special attention to *A. baumannii*. Without substantial changes in antibiotic prescribing practices, these antimicrobial-resistant bacterial infections will become increasingly difficult to treat and eliminate, and as a result, we will experience worsening health outcomes. The patients with COVID-19 that developed coinfection or secondary infection due to *A. baumannii* were predisposed to more negative outcomes, including longer hospital and ICU stays, and higher mortality. It is important to note that these outcomes are largely due to bacterial rather than viral infections. Thus, we need investment in this research, as otherwise we could face even greater negative impacts on people’s health due to MDR *A. baumannii* during current and future pandemics.
